# Infectious Aortitis Presenting as Constipation: A Diagnostic Pitfall

**DOI:** 10.7759/cureus.97129

**Published:** 2025-11-17

**Authors:** Almootazbellah M Agamy, Ahmed Musa, Nour J Al-Khasieb, Mohamed M Moustafa

**Affiliations:** 1 General Practice, Swansea Bay University Health Board, Swansea, GBR; 2 Family Medicine, Swansea Bay University Health Board, Swansea, GBR; 3 Vascular Surgery, Swansea Bay University Health Board, Swansea, GBR; 4 Accident and Emergency, Swansea Bay University Health Board, Swansea, GBR

**Keywords:** abdominal pain, aortitis, atypical back pain, constipation, unwell

## Abstract

Aortitis is an uncommon but important differential diagnosis to consider in patients presenting with vague abdominal or back pain, often posing a diagnostic challenge due to its nonspecific symptoms and clinical features. We present the case of a 59-year-old male with a two-week history of back pain radiating to the lower abdomen, initially presumed to be constipation by his primary care physician, who was later diagnosed with *Streptococcus pneumoniae*-associated infectious aortitis. At the first assessment by his general practitioner, his symptoms were mild, and he was treated with laxatives; however, he subsequently presented to the ED with severe pain requiring opioid analgesia. On examination, he was afebrile, with severe abdominal tenderness and an empty rectum on digital rectal examination. His abdomen was rigid throughout. Laboratory investigations demonstrated elevated inflammatory markers, and imaging revealed features of aortitis, prompting urgent vascular surgical intervention with the insertion of a bovine graft. This procedure was performed after one week of inpatient observation and repeat imaging, which showed progression of the initial findings, including an enlarging aneurysm and a pocket of pus. The patient remained hospitalized for approximately two weeks after surgery, including seven days in the intensive therapy unit. He later developed a para-aortic collection, which was monitored without further surgical intervention, and was discharged with two to four weeks of IV antibiotics. This case highlights how vascular pathology can mimic benign gastrointestinal conditions in the early stages, underscoring the importance of prompt imaging and multidisciplinary collaboration among family medicine, general surgery, and vascular surgery teams to ensure timely diagnosis and management.

## Introduction

Aortitis refers to inflammation of the aortic wall and can have numerous causes. It is relatively rare overall and is particularly uncommon when attributed to infectious pathogens but remains a high-risk, potentially life-threatening condition [1,2]. Noninfectious aortitis typically arises from autoimmune or inflammatory etiologies such as giant cell arteritis or Takayasu arteritis, whereas infectious aortitis (IA) most commonly results from microbial invasion of the aortic wall.

IA is uncommon in the modern antibiotic era and often poses diagnostic challenges because its symptoms are frequently nonspecific [2,3]. Known risk factors include advanced age, atherosclerosis, immunosuppression, intravascular instrumentation, or bacteremia from distant foci [3,4]. Typical clinical features may include fever, back or abdominal pain, malaise, and signs of systemic infection, although many patients present atypically [2,5]. Delayed diagnosis may lead to complications such as aneurysm formation, rupture, or sepsis [2,6]. We report a case of IA in a relatively healthy patient, in which the primary presentation mimicked other vascular or abdominal inflammatory conditions, ultimately requiring cross-disciplinary input before the correct diagnosis was made.

Although uncommon, IA must be considered in patients with unexplained systemic symptoms and vascular imaging abnormalities. The literature describes variable presentations, including some that resemble noninfectious vasculitis or aortic aneurysm without overt signs of infection. At the same time, management often requires both antimicrobial therapy and surgical or endovascular intervention [2,4]. Imaging modalities such as CT angiography, magnetic resonance angiography, PET-CT, and MRI are critical for accurate diagnosis.

This case underlines the importance of a high index of suspicion, early imaging, and a coordinated multidisciplinary approach to achieve favorable outcomes [7,8]. Notably, this case is particularly interesting because the patient presented with constipation and no fever, with further investigations and imaging initiated only when the severity of the pain was found to be disproportionate to the presenting symptoms.

## Case presentation

A 59-year-old male presented to the ED with a two-week history of progressive back and abdominal pain. Associated symptoms included constipation unresponsive to suppositories and nausea without vomiting. There were no urinary symptoms. His past medical history was notable for type 2 diabetes mellitus, and he was an active smoker.

On arrival, he appeared to be in significant pain and required opioid analgesia, including morphine. He was afebrile and hemodynamically stable, with unremarkable cardiovascular and respiratory examinations. Abdominal examination revealed diffuse tenderness without peritonism.

Initial laboratory investigations demonstrated elevated inflammatory markers (Table 1).

**Table 1:** Summary of laboratory findings on presentation.

Test	Observed value	Reference range
White cell count (× 10⁹/L)	13.2	4.0-11.0
Neutrophils (× 10⁹/L)	11	2.0-7.5
CRP (mg/L)	180	<5
Hemoglobin (g/L)	132	130-180

**Figure 1:**
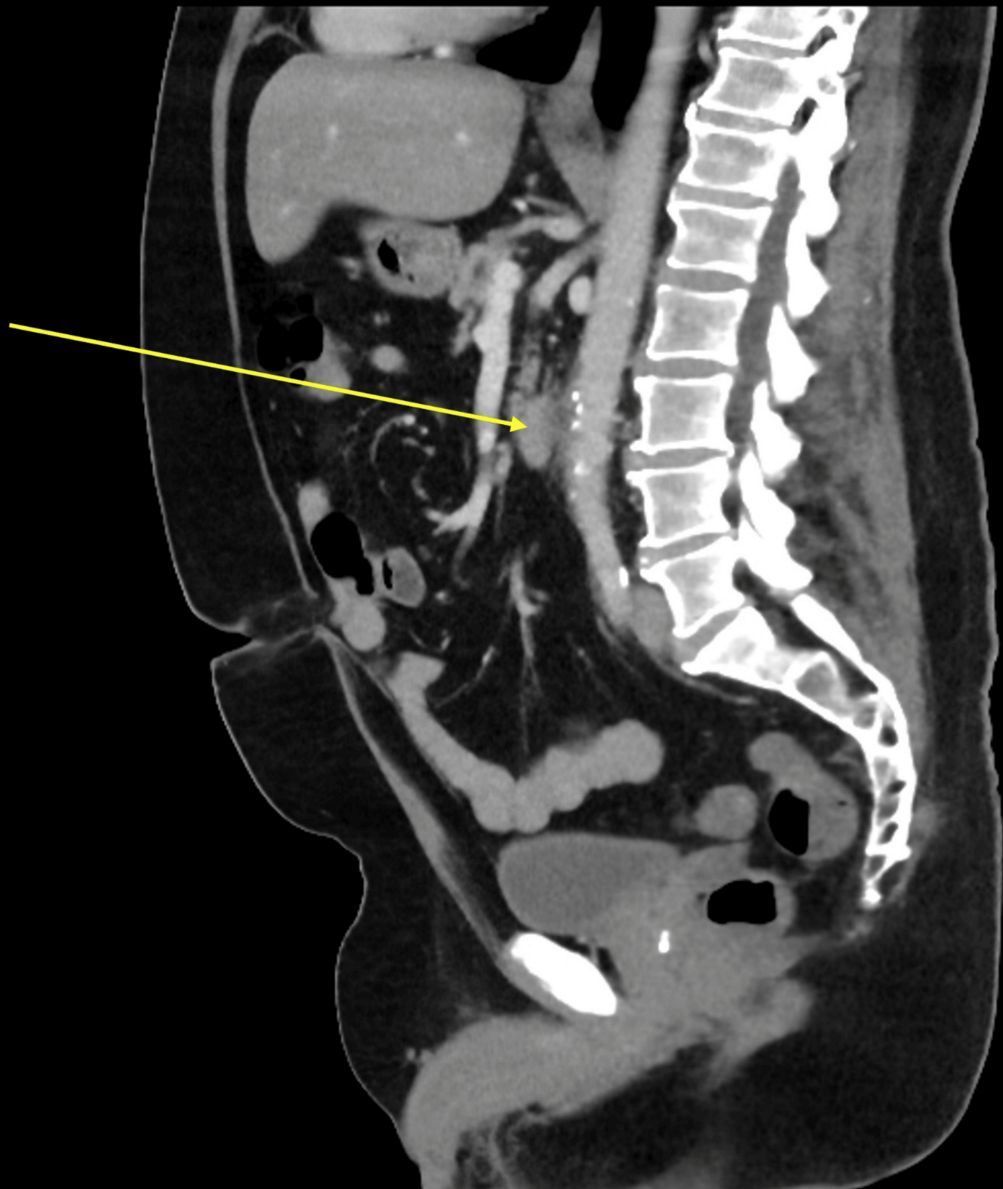
Sagittal contrast-enhanced CT of the abdomen and pelvis showing fusiform bulging of the mid-abdominal aorta with surrounding fat stranding and calcific changes, consistent with early aortitis or atherosclerotic dilatation.

**Figure 2:**
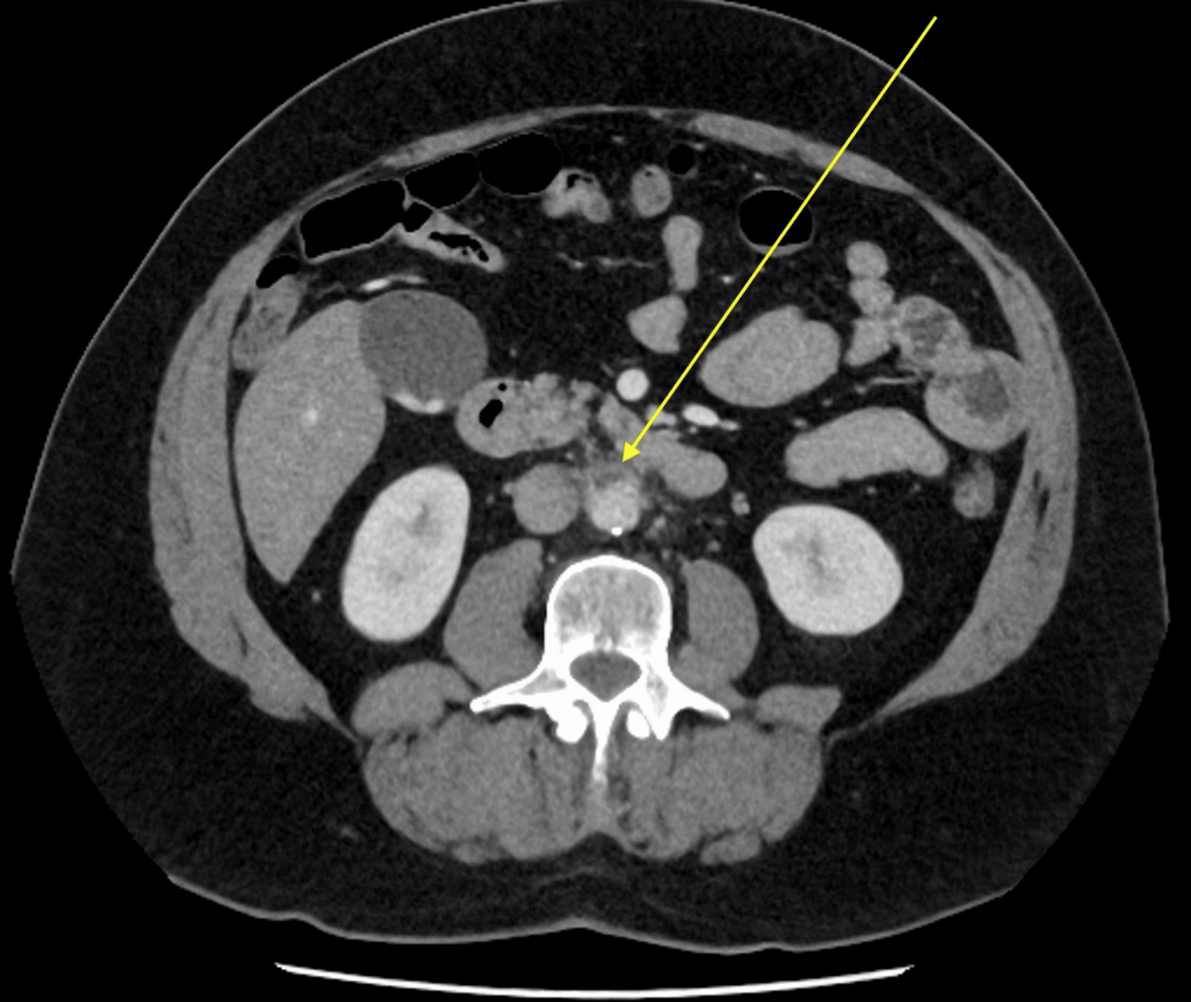
Axial contrast-enhanced CT of the abdomen and pelvis at the same level demonstrating fusiform dilatation and periaortic fat stranding, corresponding to early aortitis or atherosclerotic dilatation.

A contrast-enhanced CT scan of the abdomen and pelvis demonstrated fusiform bulging and dilatation of the mid-abdominal aorta with surrounding fat stranding and scattered calcific specks (Figure 1, Figure 2). Differential considerations at this stage included acute aortitis versus atherosclerotic dilatation. The patient was admitted under the surgical team for further review and serial imaging. The vascular team evaluated the patient at the bedside. Initially, a repeat contrast-enhanced CT of the abdomen and pelvis was recommended in one month to monitor the aortic dilatation, as the patient’s symptoms had shown slight improvement at that time.

A vascular surgery multidisciplinary team (MDT) meeting was held the following day, during which the initial plan was revised. It was agreed that a repeat CT scan should be performed within a week if the patient’s symptoms failed to improve. The infectious diseases team was consulted and recommended additional investigations to identify a potential infectious cause of the aortitis, including a full vasculitis screen; serology for syphilis, tuberculosis, and HIV; blood cultures; and an echocardiogram.

Serial blood tests during this period demonstrated rising inflammatory markers, including increasing CRP levels and neutrophil count. Based on these findings, the infectious diseases team advised commencing IV ceftriaxone as the antibiotic of choice due to its broad-spectrum coverage. The transthoracic echocardiogram revealed no evidence of infective endocarditis.

Additional results obtained during the observation period are summarized in Table 2.

**Table 2:** Further investigations.

Test	Observed value/result	Reference range
Blood cultures	No growth	N/A
Anti-nuclear antibody screen	Positive (titer 1:640)	N/A
Anti-dsDNA antibodies (IU/mL)	<9.8	0.0-26.9
Anti-ENA screen	Negative	N/A
ANCA screen	Negative	N/A
Syphilis screen	Negative	N/A
Virology – blood-borne virus screen	Negative for HIV, hepatitis B, and hepatitis C	N/A
*Brucella*complement fixation test	No serological evidence of *Brucella *infection	N/A
Beta-glucan antigen test	Negative	N/A

**Figure 3:**
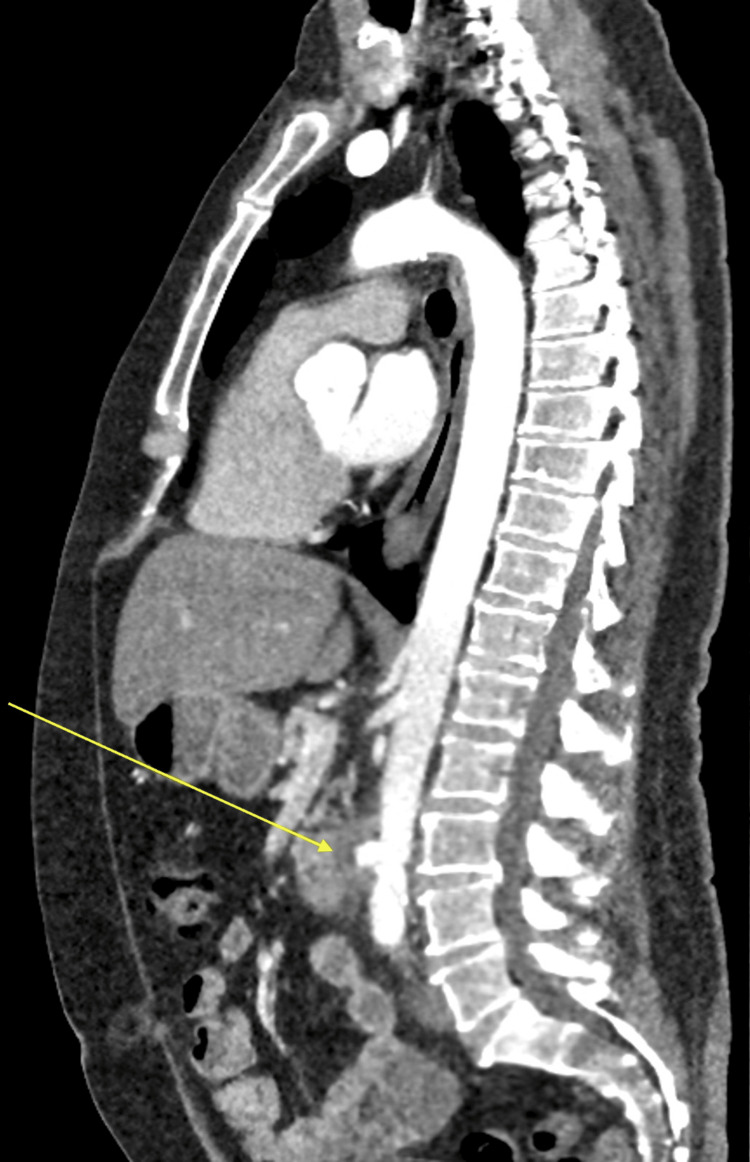
Sagittal contrast-enhanced CT of the abdomen and pelvis demonstrating progression of periaortic inflammation and anterior wall bulging of the mid-abdominal aorta, consistent with pseudoaneurysm formation.

**Figure 4:**
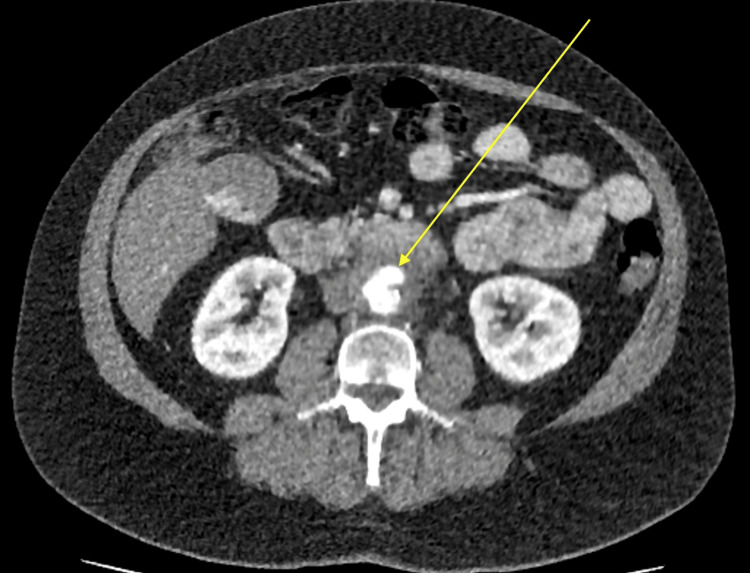
Axial contrast-enhanced CT of the abdomen and pelvis showing increased periaortic fat stranding and asymmetric anterior wall dilatation of the mid-abdominal aorta, concerning for a developing pseudoaneurysm and impending rupture.

A subsequent vascular MDT review one week into admission noted worsening pain and persistently elevated inflammatory markers. Repeat CT angiography demonstrated progression of periaortic inflammation, with increased fat stranding and anterior bulging of the mid-abdominal aorta, consistent with a developing pseudoaneurysm and suggestive of a mycotic process (Figure 3, Figure 4). Following discussion and counselling, the decision was made to proceed with open repair using a bovine pericardial patch.

Intraoperatively, infection of the infrarenal aorta was confirmed, with an anterior pseudoaneurysm and a pus-filled cavity that was contained by the duodenum. The duodenal involvement was considered reactive, with no evidence of an aortoduodenal fistula.

**Figure 5:**
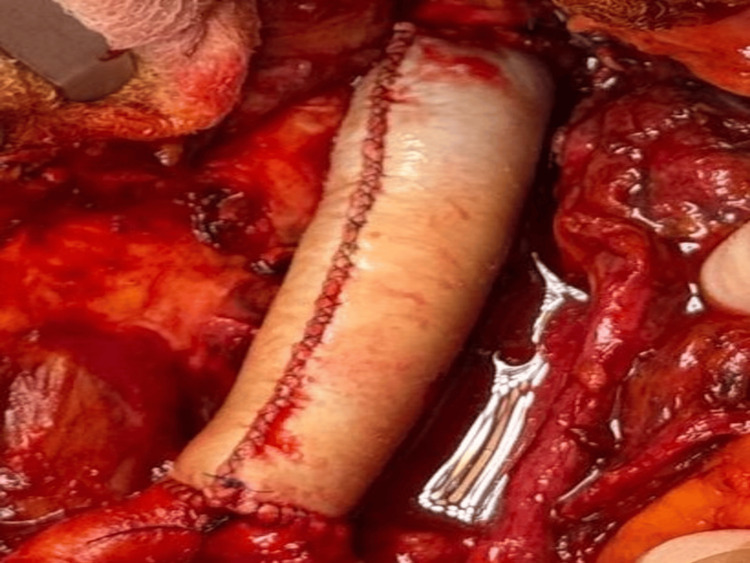
Intraoperative photograph showing placement of the bovine pericardial graft following resection of the infected infrarenal aorta. The graft is sutured in place with resolution of the pseudoaneurysm.

The affected aortic segment was resected and reconstructed using a bovine pericardial graft (Figure 5). Adjacent lymph nodes were also excised. The patient was managed postoperatively in the intensive therapy unit for seven days, during which he initially required high-flow nasal oxygen but was successfully weaned to room air. Epidural analgesia was transitioned to oral analgesia as his mobility improved. Although persistent hypertension was noted, his renal function recovered, with creatinine levels returning toward his normal baseline and pre-admission estimated glomerular filtration rate known to be within normal limits.

**Figure 6:**
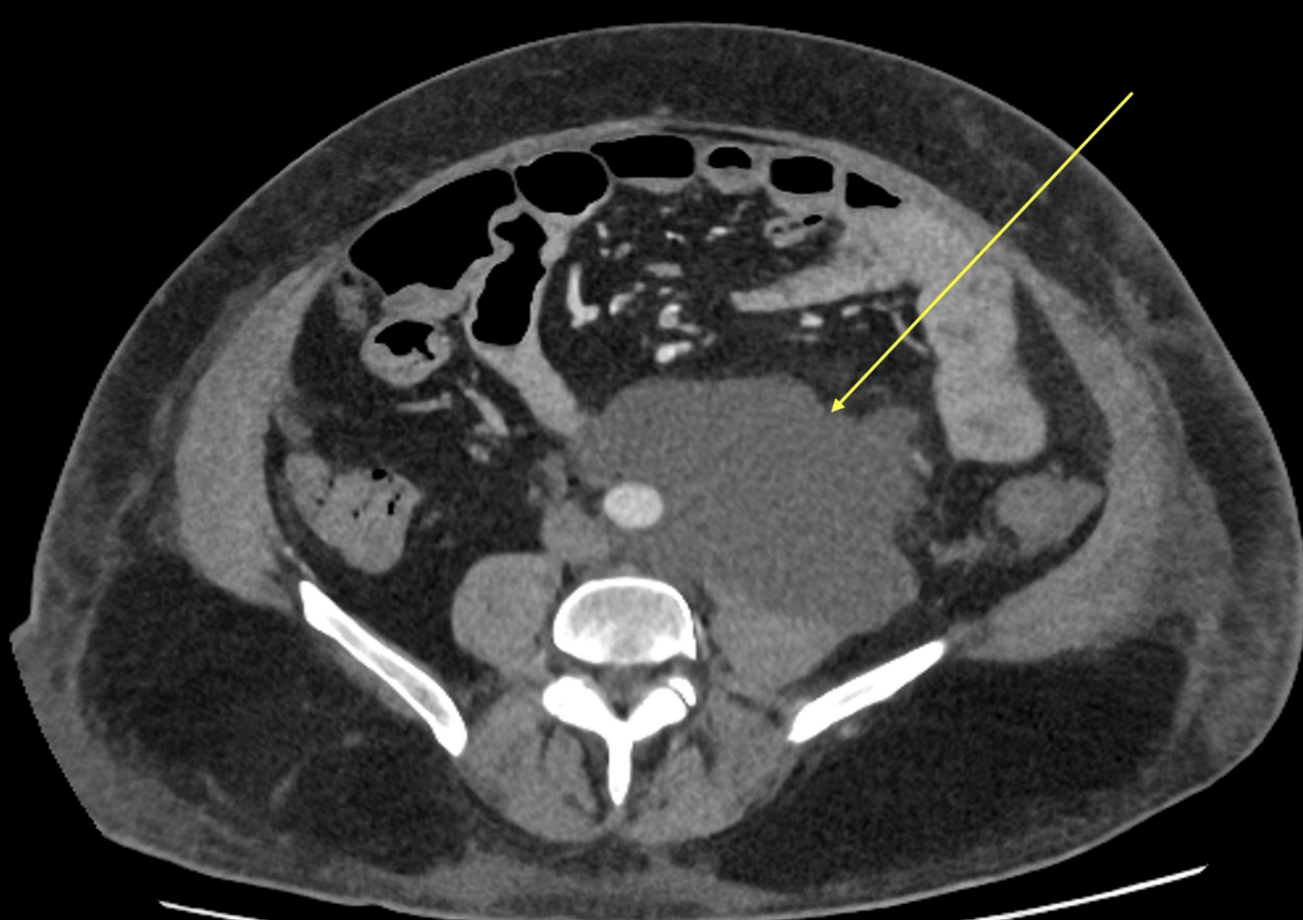
Axial CT image of the abdomen and pelvis demonstrating a large, low-attenuation fluid collection along the abdominal aorta, with no evidence of leak. The collection abuts the left psoas muscle and lies close to the left ureter, with no current ureteric obstruction.

During his subsequent ward stay, the patient developed a postoperative kidney infection, which responded well to antibiotic therapy. Later, he experienced a recurrence of abdominal and back pain. A contrast-enhanced CT scan revealed a 12-cm left para-aortic fluid collection without radiological evidence of discitis (Figure 6). MRI of the spine also showed no features of discitis or osteomyelitis (images not shown). The collection was considered consistent with a postoperative seroma or lymphocele. He was discharged with arrangements for outpatient follow-up.

The patient subsequently re-presented one week after discharge with abdominal swelling. Repeat CT imaging demonstrated a slight increase in the size of the para-aortic collection; however, his vital signs and inflammatory markers remained within normal limits. A CT urogram excluded a urinoma or ureteric leak.

Additional diagnostic results later became available. Molecular analysis of the excised aortic tissue identified *Streptococcus pneumoniae* by PCR, with concurrent detection of PBP2, indicating β-lactam resistance consistent with penicillin-non-susceptible pneumococcal infection. In light of these findings, the infectious diseases team recommended continuation of ceftriaxone and the addition of teicoplanin, administered through the outpatient parenteral antimicrobial therapy service. Teicoplanin was subsequently discontinued due to gastrointestinal intolerance and replaced with oral linezolid. The combination of ceftriaxone and linezolid was continued for a total of four weeks.

**Figure 7:**
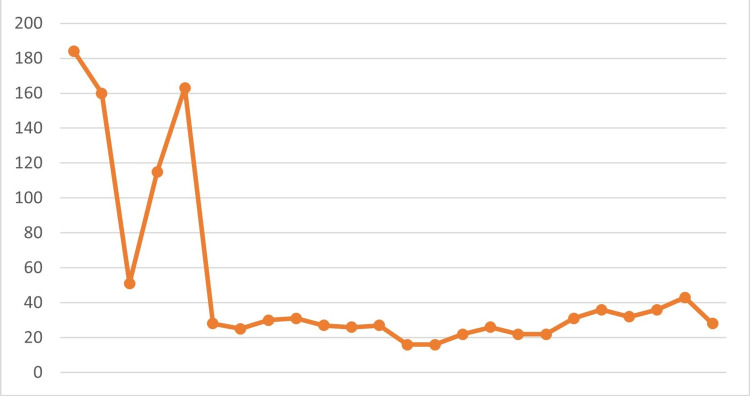
CRP trend during treatment

The patient showed a good overall clinical response, supported by a progressive decline in CRP levels throughout medical and surgical treatment (Figure 7).

At follow-up in the vascular surgery clinic, the patient appeared clinically well, had regained weight, and his renal function had returned to normal. A subsequent review in the infectious diseases clinic a few days later showed normal white cell count and CRP levels. His antibiotic therapy was therefore discontinued, and the peripherally inserted central catheter was removed. Ongoing clinical and imaging follow-up was arranged to monitor the para-aortic collection, which was presumed to be chylous in origin. Drainage would be considered if the collection increased in size or resulted in compressive symptoms, such as nausea, vomiting, or reduced appetite.

## Discussion

This case illustrates a rare and clinically significant instance of IA with mycotic pseudoaneurysm formation caused by *S. pneumoniae*. While IA itself is uncommon, its association with pneumococcal infection is even rarer in the post-antibiotic era [1,2]. The nonspecific presentation of back and abdominal pain initially prompted consideration of gastrointestinal causes, reflecting the diagnostic ambiguity of this condition, particularly as the patient’s primary complaint was constipation. The absence of fever or systemic symptoms further contributed to diagnostic uncertainty and delayed targeted management.

IA can present with a broad spectrum of intra-abdominal or vascular disorders, and diagnosis largely relies on integrating clinical suspicion with radiological and microbiological data [3]. In this case, progressive inflammatory changes surrounding the infrarenal aorta on successive CT imaging prompted urgent vascular review and guided the decision for surgical intervention. Serial imaging was pivotal in identifying the progression from mural inflammation to pseudoaneurysm formation, thereby preventing rupture through timely surgical repair.

A key feature of this case was the molecular identification of *S. pneumoniae* by PCR, with concurrent detection of PBP2 indicating β-lactam resistance. Pneumococcal aortitis is exceptionally rare and typically affects elderly or immunocompromised patients with underlying atherosclerotic disease [5,6]. Molecular diagnostics have been shown to improve pathogen detection in culture-negative IA, enabling more precise antibiotic selection and favorable outcomes [9]. In this instance, PCR provided critical microbiological clarity when conventional cultures were negative, allowing for targeted antimicrobial therapy against *S. pneumoniae *and confirming the need for prolonged β-lactam coverage.

The patient’s clinical course was further complicated by a para-aortic collection and postoperative renal infection, each requiring coordinated multidisciplinary management. Studies underscore the importance of MDT involvement, including vascular surgeons, infectious disease specialists, radiologists, and intensive care teams, to optimize decision-making and reduce morbidity. In this case, routine MDT reviews facilitated serial assessment of imaging findings, modification of antimicrobial therapy, and organized follow-up across specialties.

Definitive management of IA necessitates both surgical and medical interventions, including appropriate antimicrobial therapy. The affected aortic segment was resected and reconstructed using a bovine pericardial graft, selected based on evidence suggesting lower rates of reinfection and thrombosis compared with synthetic prostheses [10]. Postoperative antibiotic therapy was extended for four weeks, consistent with recommendations for prolonged therapy following vascular graft placement [5,6].

Long-term follow-up with serial imaging is essential, as late recurrence or graft-related infection may occur months after surgical repair. Recent reviews highlight that combining early surgical intervention with prolonged, targeted antimicrobial therapy, guided by multidisciplinary input and close imaging surveillance, offers the best survival outcomes in IA [11].

## Conclusions

This case underscores the diagnostic complexity and clinical severity of IA, particularly when conventional cultures are negative but molecular testing identifies resistant pathogens. It highlights the critical role of serial imaging, molecular diagnostics, and multidisciplinary collaboration in the timely recognition and management of such life-threatening infections. Clinicians should maintain a high index of suspicion for aortic infection in patients presenting with persistent abdominal or back pain and elevated inflammatory markers, especially those with comorbidities such as diabetes or a history of smoking. Early recognition and intervention, supported by advanced molecular techniques and coordinated multidisciplinary care, are essential to improving outcomes in this rare but potentially fatal vascular condition.
